# Copper-coordination driven nano-frameworks for efficient colorectal cancer chemo-immunotherapy by suppression of cancer cell stemness

**DOI:** 10.1016/j.mtbio.2025.101707

**Published:** 2025-03-24

**Authors:** Yichun Huang, Hailong Tian, Zhimin Yue, Lei liang, Canhua Huang, Huili Zhu, Jun Yang

**Affiliations:** a Department of Surgical Oncology, The First Affiliated Hospital of Kunming Medical University, Kunming, 650032, China; b Department of Biotherapy, Institute of Oxidative Stress Medicine, Cancer Center and State Key Laboratory of Biotherapy, West China Hospital, Sichuan University, Chengdu, 610041, China; c Frontiers Medical Center, Tianfu Jincheng Laboratory, Chengdu, 610041, China; d Department of Reproductive Medicine, Key Laboratory of Birth Defects and Related Diseases of Women and Children of Ministry of Education, West China Second University Hospital of Sichuan University, Chengdu, 610041, China

**Keywords:** Colorectal cancer, Cancer cell stemness, Cuproptosis, cGAS-STING, Chemo-immunotherapy

## Abstract

Cancer stemness, characterized by the self-renewal and differentiation capabilities of cancer stem cells (CSCs), is a critical determinant of colorectal cancer (CRC) chemo-immunotherapy. Herein, we repurposed copper-coordination driven metal-organic nano-frameworks (Cu-MOFs) to address the chemo-immunotherapy resistance posed by cancer stemness. These repurposed Cu-MOFs were loaded with the chemotherapeutic agent cisplatin (CDDP), resulting in the formation of Cu-MOF@CDDP. The Cu-MOF@CDDP are efficiently internalized by CRC cells via nanoparticle mediated endocytosis, where they release free copper ions (Cu^2+^) and CDDP in a high-glutathione (GSH) environment. After that, CDDP forms DNA-CDDP adducts that inhibit DNA synthesis and repair, while Cu^2+^ induces cuproptosis by disrupting mitochondrial metabolism. Moreover, DNA fragments originating from both the nucleus and mitochondria activate the cGAS-STING pathway, thereby initiating anti-tumor immune responses. Meanwhile, Cu^2+^ depletes intracellular GSH and induces cuproptosis, leading to the downregulation of stemness-related proteins such as ZEB1 and c-MYC, which enhances the efficacy of chemo-immunotherapy by targeting the critical pathways involved in maintaining stemness. Consequently, our results underscore the substantial promise of Cu-MOFs in overcoming stemness-driven therapeutic resistance, offering a transformative approach to sensitize chemo-immunotherapy.

## Graphical abstract

**Figure:**
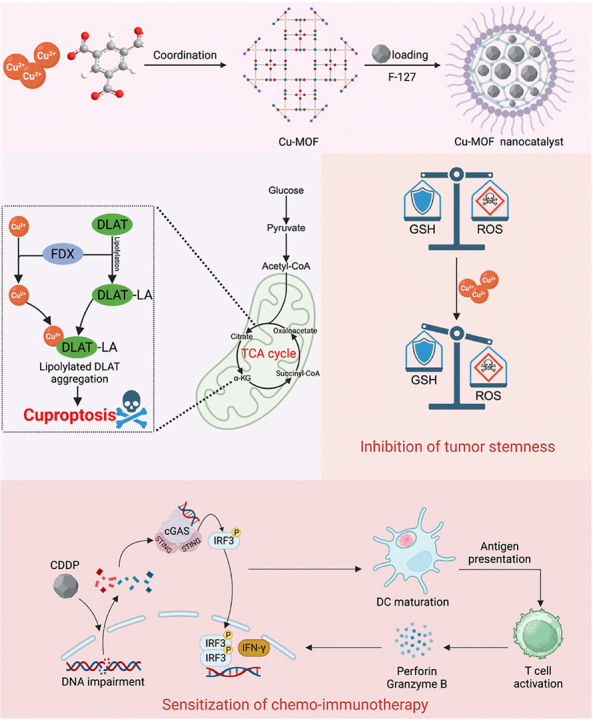

## 1 Introduction

Colorectal cancer (CRC) has been considered as the second leading cause of tumor-related mortality [[1], [2]]. Despite surgical intervention, over 30 % of CRC patients experience long-term recurrence under standard treatment regiments [3,4]. Consequently, neoadjuvant chemotherapy is often required to reduce the risk of postoperative recurrence. Cisplatin (CDDP), a widely used chemotherapeutic agent, induces DNA damage by binding to DNA bases, ultimately leading to cellular apoptosis [5,6]. Interestingly, DNA fragments resulting from CDDP-induced damage can activate the cGAS-STING signaling pathway, promoting dendritic cell (DC) maturation and subsequent T cell activation to eliminate CRC cells [7]. However, stemness-like CRC cells often evade the full impact of chemo-immunotherapy, complicating treatment outcomes [8].

The metabolic reprogramming in cancer cells elevates intracellular reactive oxygen species (ROS) levels, resulting in oxidative stress, a hallmark characteristic of tumor development [[9], [10], [11]]. In general, cancer cells with stemness-like characteristics can enhance the antioxidant defense system by up-regulating NRF2 transcription factor expression, promoting the metabolism level of glutathione (GSH), or increasing the activity of glutathione peroxidase 4 (GPX4). Hence, these cells maintain low ROS levels, resisting oxidative damage from chemotherapeutic agents like CDDP [12,13]. Taken together, the efficient inhibition of the antioxidant defense system can be acted as an important therapeutic strategy to suppress cancer cell stemness for sensitizing chemo-immunotherapy.

Notably, copper ions (Cu^2+^) has emerged as a potential therapeutic agent by inducing cuproptosis, disrupting the tricarboxylic acid cycle in mitochondrial respiration, and presenting a novel strategy to target cancer cell stemness [14]. Notably, Cu^2+^, functioning as potent peroxide-like enzymes, can induce intracellular ROS accumulation through a Fenton-like reaction, causing mitochondrial DNA impairment [15,16]. The utilization of iron ions (Fe^2+^ or Fe^3+^)-based MOFs as catalysts for the Fenton reaction in chemodynamic therapy represents a well-established paradigm, offering a highly promising oxidative stress-mediated antitumor strategy. This damaged DNA further activates the cyclic GMP-AMP synthase (cGAS)-stimulator of interferon genes (STING) pathway, resulting in proinflammatory cytokine secretion and innate immune activation [17]. However, to date, a copper-coordination-driven self-assembly nanoplatform designed to suppress cancer cell stemness remains unreported.

**Scheme 1:**
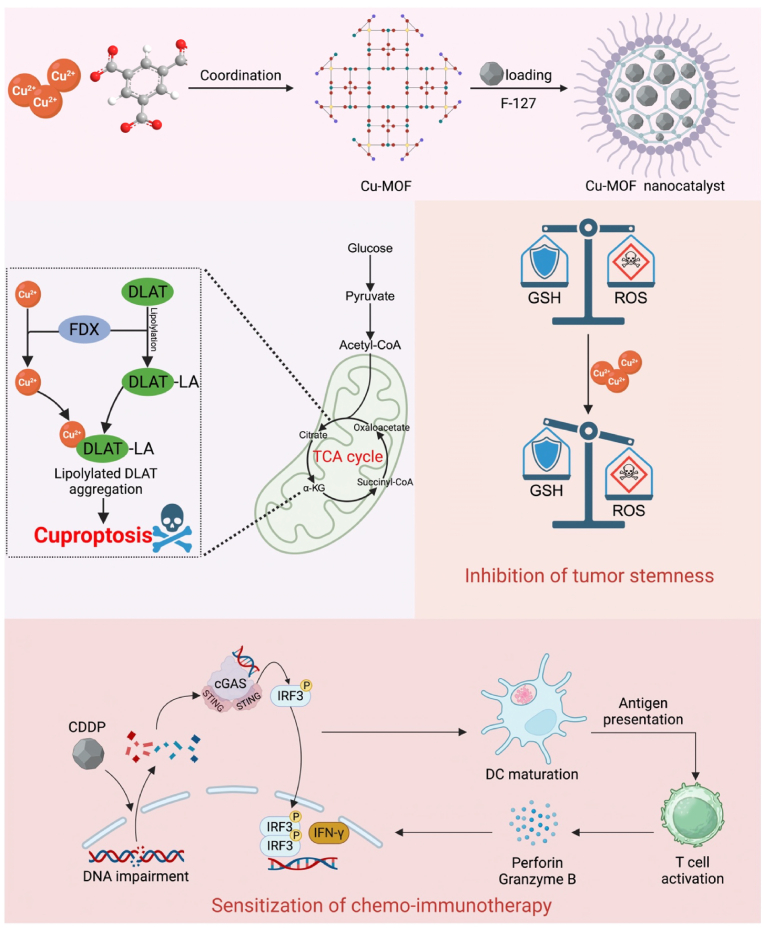
Repurposed Copper-based Metal-organic Frameworks for Efficient Suppression of Cancer Stemness and Sensitization of Chemo-immunotherapy.

Inspired by these findings, we rationally developed a Cu-coordination driven self-assembly nanoplatform to sensitize chemo-immunotherapy by targeting cancer cell stemness [18]. As shown in Scheme 1, the trimeric acid was rationally coordinated with Cu^2+^ to construct repurposed metal-organic nano-frameworks (Cu-MOFs). These Cu-MOFs were subsequently loaded with the chemotherapeutic agent cisplatin (CDDP), yielding the Cu-MOF@CDDP nanoparticles (also named as NPs). The NPs are efficiently internalized by colorectal cancer (CRC) cells through endocytosis, where they release free Cu^2+^ and CDDP in the high-GSH environment. CDDP forms DNA-CDDP adducts, inhibiting both DNA synthesis and repair, while Cu^2+^ triggers cuproptosis by disrupting mitochondrial metabolism. Additionally, DNA fragments from both the nucleus and mitochondria activate the cGAS-STING pathway, thereby initiating an innate immune response. Furthermore, Cu^2+^ depletes intracellular GSH levels and induces cuproptosis, leading to the downregulation of stemness-associated proteins such as SOX2 and c-MYC, thereby enhancing the efficacy of chemo-immunotherapy. Therefore, Cu-MOF@CDDP demonstrated significant inhibitory effects on CRC proliferation and distant metastasis without notable toxicity on major organs, offering a novel strategy to potentiate chemo-immunotherapy.

## 2 Result and discussion

### 2.1 Characterization of Cu-MOF@CDDP

**Fig. 1:**
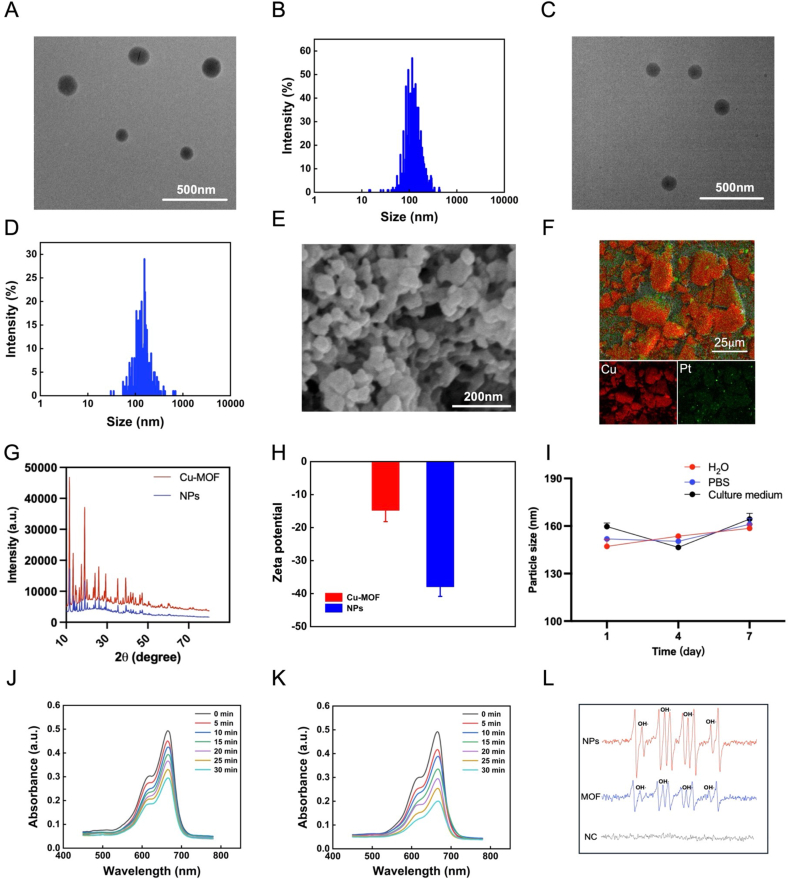
**Characterization of the Cu-MOF@CDDP Nanoparticles** A) TEM images and B) size distribution of Cu-MOF. Scale bar: 500 nm. C) TEM images and D) size distribution of the NPs. Scale bar: 500 nm. E) SEM images illustrating the morphology of the NPs. Scale bar: 200 nm. F) HAADF-STEM image of the NPs, accompanied by elemental mapping for Cu and Pt. Scale: 25μm. G) XRD patterns of the NPs and Cu-MOF, demonstrating crystallographic structure. H) Zeta potential measurements of Cu-MOF and the NPs, indicating surface charge characteristics. I) Assessment of the NPs stability over a 7-day period in aqueous environments, PBS, and culture medium. J) and K) Detection of OH• generation by Cu-MOF and the NPs, respectively, utilizing MB as a molecular probe. L) Detection OH• generation in NC, Cu-MOF and the NPs by ESR.

Transmission electron microscopy (TEM) images revealed that the self-assembly process resulted in nano-frameworks with a regular shape for the repurposed Cu-MOFs (Fig. 1A), and the particle size of Cu-MOF was approximately 110 nm (Fig. 1B). Subsequently, CDDP was encapulated in Cu-MOFs through stirring to form Cu-MOF@CDDP, which exhibited a significant morphological change in particle size and TEM images (Fig. 1C and D). Hydration particle size analysis showed that the mean size of NPs was 130 nm, which was consistent with the size of enhanced penetration and retention (EPR) effect for passive targeting delivery. The increase in particle size from Cu-MOFs to Cu-MOF@CDDP suggested the efficient loading of CDDP. Nanoparticle morphology was characterized by field-emission scanning electron microscopy (FE-SEM, Fig. 1E), while the elemental composition and distribution were analyzed using aberration-corrected high-angle annular dark-field scanning transmission electron microscopy (HAADF-STEM, Fig. 1F) equipped with energy-dispersive X-ray spectroscopy (EDS, Figs. S1A–B, Supporting Information). The simultaneous detection of characteristic Cu and Pt signals through elemental mapping, along with their spatial colocalization within the nanostructure, unequivocally demonstrates the successful encapsulation of CDDP within the Cu-MOF matrix. Moreover, we employed X-ray diffraction (XRD) to meticulously examine the crystalline architecture of the NPs (Fig. 1G). The resultant diffraction patterns provide conclusive evidence regarding the phase purity and crystallographic properties inherent to the nanomaterial under investigation. The significant reduction of zeta potential in the NPs may be due to the large number of carboxyl groups in the structure of Pluronic F127 (Fig. 1H). Such a negative zeta potential should endow Cu-MOF@CDDP with excellent stability (Fig. 1I) [19,20]. Additionally, the specific UV–vis absorbance spectra of the repurposed Cu-MOF solution confirmed successful preparation (Fig. S1C, Supporting Information).

The repurposed Cu-MOFs mediated ROS accumulation and GSH consumption were considered as crucial steps for the suppression of cancer cell stemness. Therefore, a methylene blue (MB) probe was further utilized for evaluate the chemodynamic efficacy of Cu-MOFs. In brief, it was proposed that in the presence of GSH, the Cu-MOF@CDDP would disassemble to release CDDP and Cu-MOF fragments, subsequently generating hydroxyl radicals (OH•) that can induce the death of cancer cells by reacting with the elevated levels of H_2_O_2_ found within these cells. To mimic the tumor microenvironment, Cu-MOFs were treated with GSH and H_2_O_2_. The MB probe specifically interacts with OH•, leading to the ongoing consumption of MB. Thus, the reduction in the remaining MB concentration serves as an indirect indicator of OH• production, reflecting the chemodynamic efficacy. As illustrated in Fig. 1J–K and **S1D-E (**Supporting **Information)**, the exposure of Cu-MOFs and NPs to GSH resulted in a significant reduction in the residual MB concentration, indicative of its robust capacity to generate OH• and facilitate ROS accumulation. Additionally, as shown in Fig. 1L, the generation of OH• was detected by Electron spin resonance (ESR) and also obtained similar results. Furthermore, the repurposed Cu-MOFs exhibited pronounced sensitivity to GSH and demonstrated targeted degradation within the tumor microenvironment (TME), underscoring its promising potential for achieving effective chemodynamic therapy. Meanwhile, Cu-MOFs can be degraded by GSH to release Cu^+^ inside cancer cells, which is essential for disturbing redox balance in modulating cancer cell stemness. Consequently, we further used DTNB to quantitatively evaluate the Cu-MOFs-induced GSH consumption. DTNB reacted with GSH to form 5-mercapto-2-nitrobenzoic acid, which exhibited a maximum ultraviolet absorption at 412 nm. A reduction in absorption intensity at this wavelength suggested the lower GSH levels. Consequently, the depletion of GSH was quantified by assessing changes in absorbance. The findings shown in Fig. S1F demonstrated that the NPs decreased significantly GSH content, supporting their exceptional responsiveness to GSH and great potential in disturbing redox balance to inhibit cancer cell stemness for potentiating chemo-immunotherapy.

### 2.2 Cu-MOF@CDDP mediated endocytosis and cytotoxicity

**Fig. 2:**
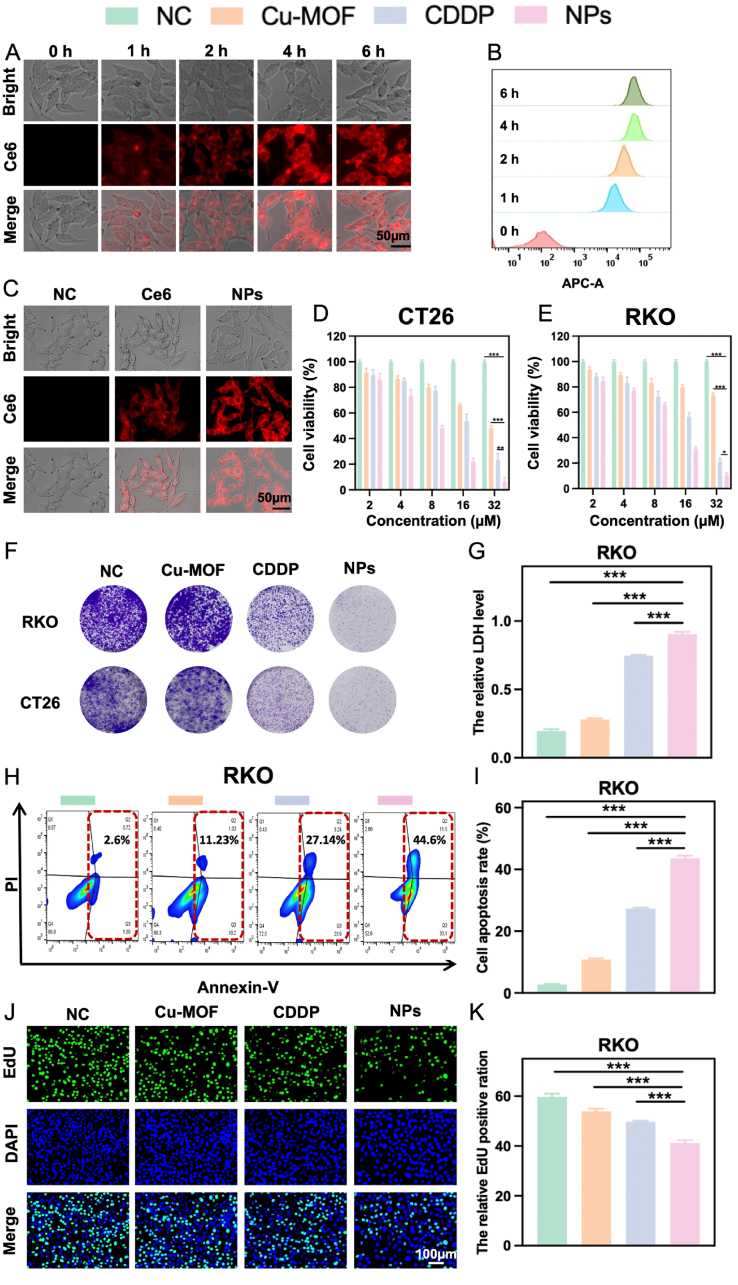
**The cellular uptake and cytotoxicity of Cu-MOF@CDDP** A) Fluorescence microscopy images and B) flow cytometry data demonstrated the cellular uptake of the NPs in RKO cells at various time points. Scale bar: 50 μm. C) Quantitative results of cellular uptake of NC, Ce6, and NPs by RKO cells following a 4-h incubation period. Scale bar: 50 μm. NC represents the Negative Control group. Cell viability of CT26 D) and RKO E) cells treated with NC, Cu-MOFs, CDDP, and NPs. F) Results of the colony formation assay for RKO and CT26 cells. G) Relative LDH release analysis in RKO cells. H) Representative flow cytometry dot plots demonstrating apoptosis profiles in RKO cells, with corresponding quantitative analysis. I) Statistical quantification of apoptotic cell populations in RKO cells, expressed as percentage of total cells. J) EdU assay results assessing proliferation in RKO cells. Scale bar: 100 μm. K) Quantitative results of EdU assay in RKO cells. The differences were considered significant for *p* values ∗ <0.05, ∗∗ <0.01, and ∗∗∗ <0.001.

For determining the optimal time for Cu-MOF@CDDP treatment, the cellular endocytosis efficiency was evaluated in various CRC cells (such as CT26 and RKO) at predesigned time intervals. Fig. 2A and B and **S2A-B** (Supporting Information) demonstrated a time-dependent cellular internalization characteristic following the observed time periods. Notably, a significant drug uptake was detected after 4 h of incubation with Cu-MOF@CDDP, suggesting that this was the optimal time point for drug treatment in subsequent *in vitro* assays. Meanwhile, we compared the internalization ability of nanoparticles with free drugs after 4 h of incubation in CT26 and RKO cells (Figs. 2C and S2C, Supporting Information). The stronger red fluorescence signal observed in cells treated with NPs indicated superior cellular internalization efficiency compared to free drugs, likely due to the more efficient nanoparticle-mediated endocytosis as opposed to passive diffusion.

Subsequently, the *in vitro* CRC efficacy of the repurposed Cu-based nanoplatform was evaluated using the MTT assay. Notably, the NPs demonstrated significantly enhanced tumor inhibition compared to both free CDDP and Cu-MOFs at equivalent doses in the aforementioned cancer cell lines (Fig. 2D and E). This superior suppression of CRC cells may be attributed to the improved cellular uptake efficiency of the NPs. Additionally, a reduction in colony formation, as illustrated in Fig. 2F, revealed a pronounced inhibition of CRC cell growth across various treatment groups, with the most potent effects observed in the NPs-treated groups. Furthermore, the anti-proliferative activity of the NPs was corroborated by lactate dehydrogenase (LDH) release assays (Fig. 2G and S2D, Supporting Information), apoptosis assays (Fig. 2H–I and S2E-F, Supporting Information), and EdU incorporation assays (Fig. 2J–K and S2G-H, Supporting Information), all of which aligned with the aforementioned findings. Collectively, these results underscore the significant potential of our developed NPs in effectively suppressing CRC progression.

### 2.3 Cu-MOF@CDDP-mediated stemness inhibition and *In Vitro* Immune Activation

**Fig. 3:**
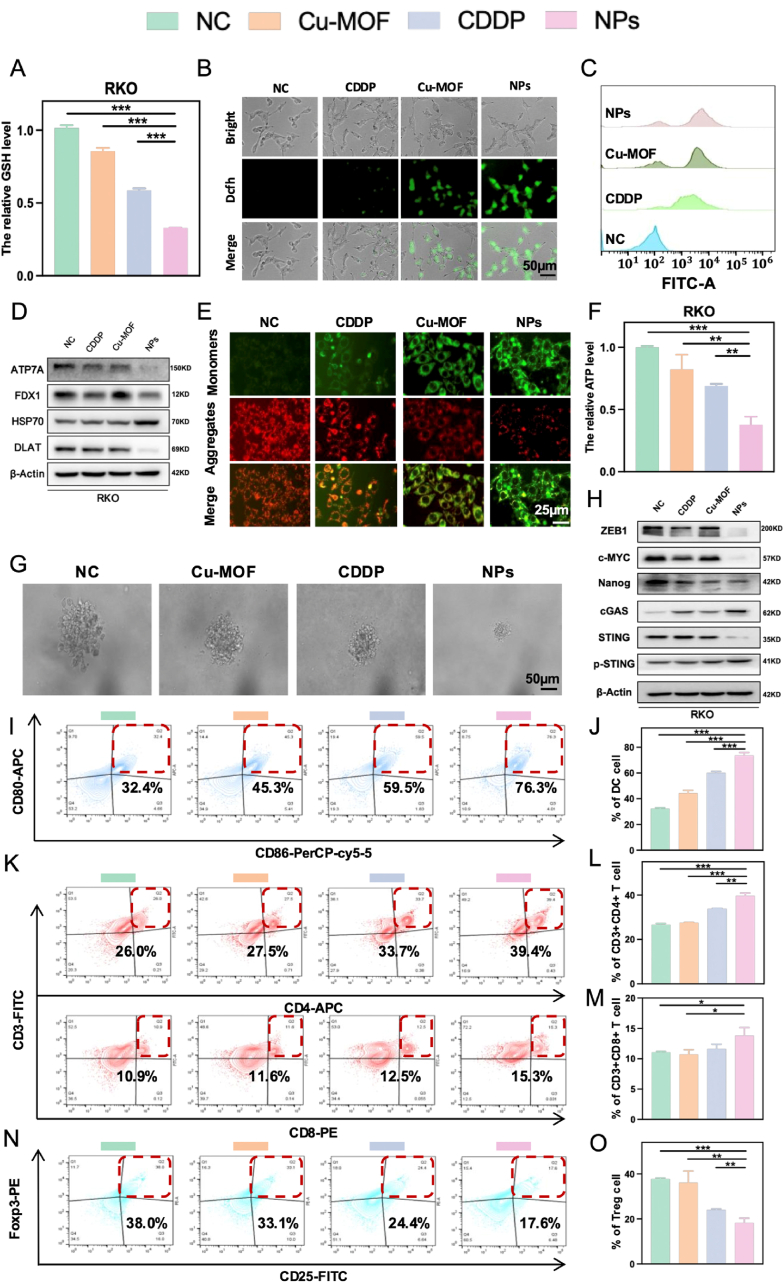
**Cuproptosis-mediated Stemness inhibition and *In Vitro* Immune Activation** A) Relative GSH analysis in RKO cells. B) Fluorescence imaging and C) Flow cytometry analysis of intracellular ROS levels in RKO cells using DCFH-DA as a probe. Scale bar: 50 μm. D) Western blot analysis of ATP7A, FDX1, HSP70, DLAT expression in RKO cells following various treatments. E) The mitochondrial membrane potential was determined by JC-1 assay with different inventions. Scale bar: 25 μm. F) Relative ATP content analysis of RKO cells. G) Sphere-formation assay results for RKO cells after different treatments. Scale bar: 50 μm. H) Western blot analysis of ZEB1, c-MYC, Nanog, c-GAS, STING, p-STING expression in RKO cells following various treatments. Flow cytometry plots and quantification results of (I–J) DCs, (K–M) CD3^+^CD4^+^/CD3^+^CD8^+^ T cells and (N–O) Tregs. The differences were considered significant for *p* values ∗ <0.05, ∗∗ <0.01, and ∗∗∗ <0.001.

The elevated Cu^2+^ content in CRC cells resulted in a significant depletion of GSH (Fig. 3A and S3A-C, Supporting Information), initiating a Fenton-like reaction that exacerbated redox imbalance (Fig. 3B–C and S3D-E, Supporting Information). Concurrently, the downregulation of FDX1, DLAT, and ATP7A, along with the upregulation of HSP70, further confirmed that the NPs effectively induced cuproptosis (Fig. 3D and S3F, Supporting Information), a process likely contributing to mitochondrial dysfunction and the suppression of cancer cell stemness. To investigate mitochondrial impairment, the fluorescent dye JC-1, a widely used physiological marker for assessing mitochondrial function, was employed to monitor changes in mitochondrial membrane potential. Typically, JC-1 forms J-aggregates that emit red fluorescence in healthy mitochondria, whereas it produces green-fluorescent monomers in damaged mitochondria. As depicted in Fig. 3E and S3G (Supporting Information), RKO and CT26 cells treated with the NPs exhibited a marked decrease in red fluorescence from J-aggregates and a corresponding increase in green fluorescence from monomers. These observations support the notion that the NPs induced ROS accumulation and cuproptosis, ultimately leading to mitochondrial damage. Furthermore, a significant reduction in intracellular ATP levels provided additional evidence of mitochondrial dysfunction (Fig. 3F and S3H, Supporting Information). Collectively, these findings highlight the mechanistic role of the NPs in disrupting mitochondrial integrity and impairing cancer cell stemness through cuproptosis induction.

Notably, recent studies have emphasized the potential of copper-based therapies to selectively target cancer cell stemness by inducing cuproptosis, a form of regulated cell death driven by Cu^2+^ overload [21]. Cancer cell stemness, which heavily relies on mitochondrial function, is particularly vulnerable to mitochondrial disruption [22]. As anticipated, the NPs effectively triggered cuproptosis and significantly reduced the formation of mammospheres (Fig. 3G and S3I, Supporting Information). Concurrently, the expression of stemness-related proteins, including ZEB1, c-MYC, and Nanog, was markedly downregulated following treatment with the repurposed Cu-MOFs (Fig. 3H and S3J, Supporting Information). These findings underscore the ability of the NPs to disrupt cancer cell stemness and highlight their potential as a therapeutic strategy for targeting cancer stem cells.

There is abundant evidence to demonstrate that cytosolic double-stranded DNA (dsDNA) originating from damaged cancer cells can directly promote the activation of the cGAS-STING pathway, thereby provoking the antitumor immunity [23]. As displayed in Fig. 3H, the activated cGAS-STING pathway suggested that the repurposed Cu-MOFs treatment efficiently controlled the cancer cell stemness for sensitizing chemo-immunotherapy. Subsequently, we developed a co-culture transwell model to investigate the immune activation potential of Cu-MOF@CDDP in relation to immune cells isolated from the spleens of BALB/C mice. As presented in Fig. 3I and J, CT26 cells treated with the NPs enhanced the maturation of dendritic cells (DCs) to the highest level compared to other groups. CD8^+^ T cells, commonly considered as cytotoxic T lymphocytes, can directly trigger cancer cell death by releasing cytotoxic molecules [24]. Additionally, CD4^+^ T cells, commonly named as helper T cells, are essential for regulating the adaptive immunity [25]. As anticipated, the NPs substantially increased the levels of CD4^+^/CD8^+^ T cells (Fig. 3K–M). Furthermore, the NPs treatment decreased the proportion of regulatory T (Treg) cells from 37 % to 19 %, indicating that Cu-MOF@CDDP may have the capacity to counteract Treg cell-mediated immune evasion (Fig. 3N and O). Taken together, the NPs could effectively sensitize chemo-immunotherapy by the suppression of cancer cell stemness.

### 2.4 The enhancing efficacy of the NPs in chemo-immunotherapy sensitization

**Fig. 4:**
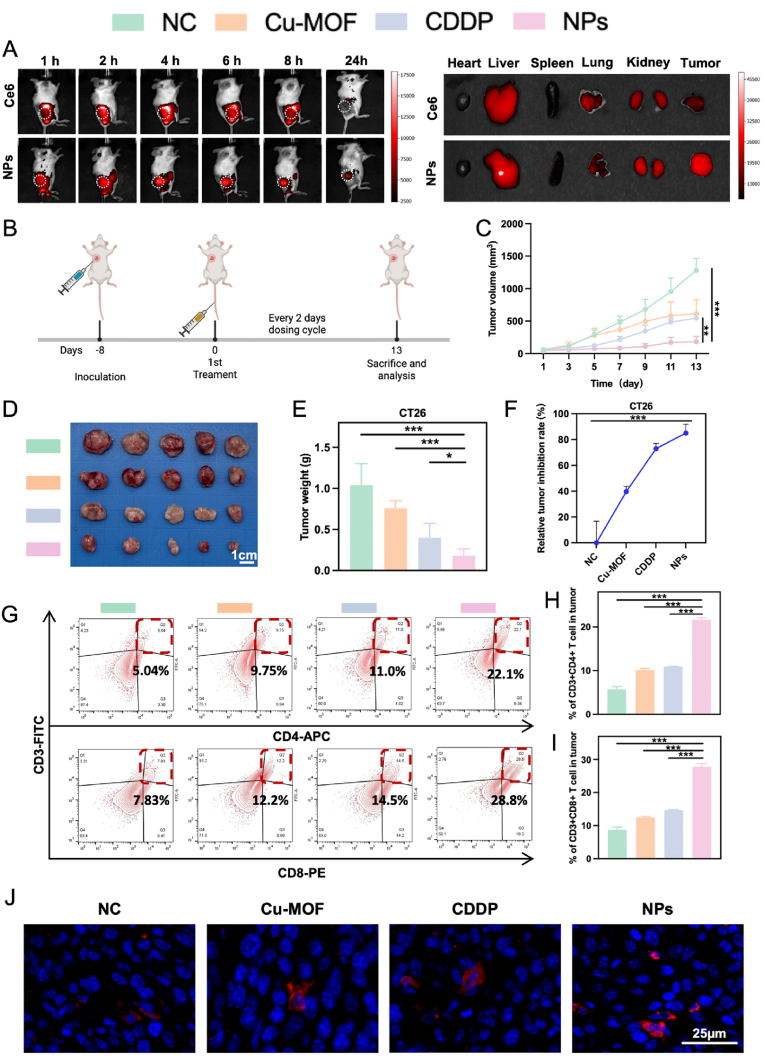
**The Chemo-Immunotherapy Sensitization of Cu-MOF@CDDP** A) Temporal *in vivo* biodistribution profiles and organ-specific accumulation patterns of the NPs following administration in mice. B) Schematic representation of *in vivo* Cu-MOF@CDDP treatment in the unilateral CRC model. C) Tumor volume change curves. D) Corresponding tumor images in different groups. Scale bar: 1 cm. E) The tumor mass and F) corresponding inhibition rate (n = 5). Flow cytometry plots and quantification of G-I) CD3^+^CD4^+^/CD3^+^CD8^+^ T cells. J) Representative immunofluorescence images depicting CD8^+^ T cells infiltration in tumor tissues. The differences were considered significant for *p* values ∗ <0.05, ∗∗ <0.01, and ∗∗∗ <0.001.

Longitudinal small-animal *in vivo* imaging studies demonstrated significantly improved tumor-targeting efficiency and prolonged retention of the NPs at the tumor site compared to free drug formulation at 24 h post-injection, as shown in Fig. 4A, suggesting enhanced tumor accumulation and stability profiles of the nanoformulation. As shown in Fig. 4B, we established a subcutaneous tumor xenograft model to study the potential of the NPs in enhancing chemo-immunotherapy sensitivity *in vivo.* Specifically, CT26 cells were injected subcutaneously into BABL/c mice, and once the xenograft tumors were stably formed, different experimental groups were subjected to drug treatments. Tumor size and mass were measured, body weight changes were monitored, and tumor growth inhibition during treatment was observed to comprehensively assess the chemosensitization effect of the NPs. Upon completion of all treatments, tumors were excised, photographed, and weighed. The results presented in Fig. 4C–E demonstrated that CDDP, Cu-MOFs, and the NPs effectively inhibited tumor growth, with the NPs treatment exhibiting the highest efficacy. It should be noted that the related tumor inhibition ratio was highest in the group of the NPs (Fig. 4F), which could be attributed to sensitized chemo-immunotherapy.

Next, we examined the extent of immune cell infiltration within the tumor tissues. As illustrated in Figs. S4A–B (Supporting Information), treatment with the NPs enhanced the presence of DCs within the tumor tissues, consistent with the findings from the *in vitro* co-culture experiments. Previous studies have shown that antigen-specific T cell (CD4^+^/CD8^+^) infiltration and activation in the TME can significantly bolster tumor inhibition by promoting intercellular immune responses. Consequently, we investigated the infiltration and activation of CD4^+^/CD8^+^ T cells in tumor tissues. As shown in Fig. 4G–I, the NPs treatment groups exhibited increased T cells (CD4^+^/CD8^+^) infiltration in tumor tissues compared to other treatment groups. Quantitative analysis of tumor-infiltrating CD8^+^ cytotoxic T lymphocytes (CTLs) revealed significantly enhanced accumulation in the NPs-treated groups compared to controls, as demonstrated in Fig. 4J. Meanwhile, the proportion of Treg cells was significantly reduced (Figs. S4C–D). Similarly, Cu-MOF@CDDP significantly increased the proportions of DCs and T cells, while markedly reducing the proportion of Treg cells in spleens (Figs. S4E–K, Supporting Information). Taken together, our designed Cu-MOF@CDDP showed great potential for sensitizing CRC chemo-immunotherapy.

### 2.5 Enhanced Immunotherapeutic Efficacy in Bilateral Tumor-Bearing Murine Models

**Fig. 5:**
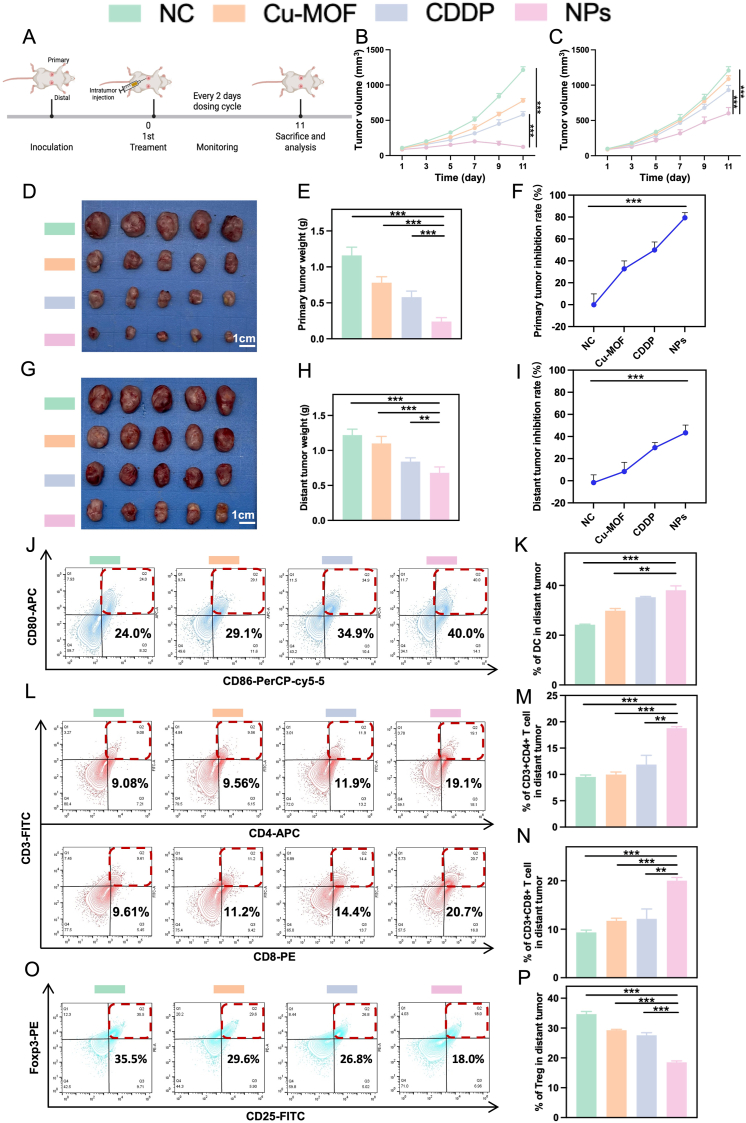
**Enhanced Immunotherapeutic Efficacy in Bilateral Tumor-Bearing Murine Models** A) Schematic illustration of the therapeutic regimen in bilateral tumor-bearing mouse model. B) Primary tumor volume change curves (n = 5). C) Distant tumor volume change curves (n = 5). D) Representative photographic images of excised primary tumors. Scale bar: 1 cm. E) Quantitative analysis of primary tumor mass and (F) corresponding tumor growth inhibition rates (n = 5). G) Representative photographic images of excised distant tumors. Scale bar: 1 cm. H) Quantitative analysis of distant tumor mass and (I) corresponding tumor growth inhibition rates (n = 5). Flow cytometry plots and quantification of J-K) DCs, L-N) CD3^+^CD4^+^/CD3^+^CD8^+^ T cells and O-P) Tregs in distant tumor. The differences were considered significant for *p* values ∗ <0.05, ∗∗ <0.01, and ∗∗∗ <0.001.

The above results have supported that the repurposed Cu-MOF nanoplatform can trigger a potent immune response by the suppression of cancer cell stemness. Based on this, we further studied the possibility of whether such a sensitized antitumor immunity could efficiently control the progression of both in situ tumors and distal metastases. Therefore, we constructed a bilateral CRC cancer model for evaluating the effects of repurposed Cu-MOF nanoplatform (Fig. 5A). Briefly, the right sites were marked as the in situ tumors, while the opposite sites were artificial models of pre-existing metastatic lesions without any treatments. The intravenous injection can accumulate drugs into the bilateral tumors, so we only performed intratumoral administration for the primary tumor to evaluate whether the immune response can be efficiently sensitized to eliminate the distant cancer cells. These mice were received an equivalent dosage of CDDP (3 mg/kg) via intratumoral administration (only in situ tumors) every two days for two weeks. After the treatment of Cu-MOF@CDDP, the size and mass of bilateral tumors were significantly suppressed (Fig. 5B–I). It should be noted that the correspond tumor inhibition ratios were consistent for the previous results, which also supported the sensitized immunotherapy against CRC (Fig. 5F and I). As shown in (Fig. 5J–K and **S5A-B)**, the NPs increased the proportion of activated DCs to in bilateral distant tumors, consistent with the data of immune co-culture. Infiltration and activation of cytotoxicity T cells in the TME could effectively eliminate cancer cells by triggering the anti-tumor immunity [26]. Flow cytometry was further conducted to assess T cell infiltration in tumor tissues. As illustrated in Fig. 5L–P and **S5C-G** (Supporting Information), the NPs demonstrated a higher population of both CD3^+^CD4^+^ and CD3^+^CD8^+^ T cells and a lower population of Treg cells infiltrated in the bilateral tumors in comparison with other groups, highlighting their significant potential for eradicating bilateral tumors through the activated anti-tumor immunity. Additionally, we also analyzed the proportion of immune cells in the spleen. Similar to the immune infiltration observed in the tumor tissues, Cu-MOF@CDDP significantly increased the proportions of DCs and T cells, while markedly reducing the proportion of Treg cells (Figs. S6A–G, Supporting Information).

Comprehensive biochemical analysis revealed that serum concentrations of hepatic function indicators (aspartate aminotransferase [AST] and alanine aminotransferase [ALT]) and renal function markers (blood urea nitrogen [BUN] and creatinine [CRE]) remained within physiological ranges in all treatment groups (Figs. S7A–D, Supporting Information), demonstrating the absence of significant nephrotoxicity or hepatotoxicity. Moreover, longitudinal monitoring of body weight in tumor-bearing mice showed stable growth curves across all experimental groups, with no statistically significant differences compared to healthy controls (p > 0.05), in contrast to the progressive weight loss observed in the blank control group (Fig. S7E, Supporting Information). Additionally, systematic safety evaluation demonstrated that the nanoformulation maintained a hemolysis rate below the acceptable threshold of 5 % (Fig. S7F, Supporting Information), fulfilling the requirements for intravenous injectable formulations. Histopathological assessment of vital organs, including cardiac, hepatic, splenic, pulmonary, and renal tissues, revealed well-preserved tissue architecture without signs of inflammation, necrosis, or other pathological alterations in nanoparticle-treated animals, comparable to saline-treated controls (Fig. S7G, Supporting Information). These findings collectively confirm the exceptional biocompatibility and biosafety of our engineered delivery platform for systemic administration.

## 3 Experimental section

### 3.1 Materials

Cu_2_(NO_3_)_2·_3H_2_O (Purity, 99.5 %), triethylamine (Purity, 99.5 %), trimesic acid (Purity >98 %), and N,N-dimethylformamide (DMF, Purity 99.8 %) were obtained from Dalian Meilun Biotech Co., Ltd. Ethanol (Purity, 99.7 %), dichloromethane, and normal saline were provided by China National Pharmaceutical Group Co., Ltd. Methylene blue (MB), 5,5′-Dithiobis-(2-nitrobenzoic acid) (DTNB), annexin V-FITC/PI apoptosis detection kit, 30 % H_2_O_2_, glutathione (GSH), thiazolyl blue (MTT), collagenase I, and red blood cell lysis solution were purchased from Solarbio (Beijing, China). The GSH cell-based detection kit, DCFH-DA assay kit, HRP-labeled Goat Anti-Rabbit and Anti-Mouse IgG (H + L) were procured from Beyotime Biotechnology. Additionally, the copper assay kits, pyruvic acid colorimetric assay Kit, succinate dehydrogenase (SDH) activity assay kit, and α-ketoglutarate (α-KG) fluorometric assay kit were obtained from Elabscience Biotechnology Co., Ltd.

### 3.2 Preparation of Cu-MOF

For the synthesis of Cu-MOF, we referred relevant literature and made minor modifications [21]. Firstly, we dissolved trimesic acid (250.0 mg) and Cu_2_(NO_3_)_2·_3H_2_O (287.5 mg) separately in two identical 6 mL mixtures of ethanol, DMF, and water (*v*:*v*:*v*, 1:1:1). Then, we mixed the two solutions and slowly added triethylamine (250 mL). After stirring well, the mixture was subjected to intermittent ultrasonication at 300 W for 1 h (sonicate 2 s, pause 3 s). Following centrifugation, the obtained raw Cu-MOF was washed twice with water and then three times with DMF to remove any free materials, after which it was soaked in a certain amount of DMF for 12 h. Subsequently, the product was centrifuged to remove DMF, and the precipitate was washed 3 times with CH_2_Cl_2_, followed by soaking the precipitate in CH_2_Cl_2_ for 1 d. Finally, the precipitate was collected by filtration, and the Cu-MOF was obtained by drying in a vacuum.

### 3.3 Preparation of Cu-MOF@ CDDP

For the preparation of Cu-MOF@CDDP, Cu-MOF and CDDP were dispersed in ethanol and stirred at room temperature (1000 rpm) for 24 h. Then we obtained Cu-MOF@CDDP by centrifugation and washed the product three times with ethanol to remove any free CDDP, and finally dried it under vacuum. The loading amount of CDDP in Cu-MOF@CDDP can be calculated using the following equation:

$$wt\%=\left(1-\frac{A_{\text{CDDP}\;\text{in}\;\text{the}\;\text{supernatant}}}{A_{\text{CDDP}\;\text{before}\;\text{loading}}}\right)\times \frac{M_{\text{CDDP}}}{M_{\text{Cu}-\text{MOF}\;@\;\text{CDDP}}}$$

In the equation, $A_{\text{CDDP}\;\text{in}\;\text{the}\;\text{supernatant}}$ represents the absorbance of the supernatant, and $A_{\text{CDDP}\;\text{before}\;\text{loading}}$ represents the absorbance before the loading of CDDP, $M_{\text{CDDP}}$ represents the initial mass of CDDP, and $M_{\text{Cu}-\text{MOF}@\text{CDDP}}$ represents the mass of Cu-MOF @ CDDP.

### 3.4 Preparation of repurposed Cu-MOF nanoplatform

The classic thin-film dispersion method was used to synthesis of Cu-MOF NPs. Firstly, 5 mg Cu-MOF and 10 mg Pluronic F127 were mixed with ethanol and vigorously stirred (1000 rpm) for 15 min to dissolve. Then, the ethanol in the solution was removed by evaporation. Subsequently, 5 mL of deionized water was added to the reaction vessel and sonicated the mixture for 30 min. Finally, Cu-MOF NPs solution with a concentration of 1 mg/mL was obtained. Additionally, we prepared Cu-MOF@CDDP NPs using the same method.

### 3.5 The characterizations of Cu-MOF@CDDP

The characterizations of Cu-MOF and Cu-MOF@CDDP were recorded using a TEM (JEM-200CX) and a Malvern zeta sizer nano analyzer.

### 3.6 The detection of Fenton-like reaction

We investigated the generation of OH• in Cu-MOF@CDDP NPs using the spectroscopic method of methylene blue (MB). The specific method was diluted the Cu-MOF@CDDP NPs from 1 mg/mL to 300 μg/mL with distilled water. Then we added 1 mL 20 mM GSH to 1 mL Cu-MOF@CDDP and incubated for 0.5 h. We also set up a blank control group without GSH. Then, we added MB and H_2_O_2_ to both groups of solutions respectively, with final concentrations of 10 mg/mL and 10 mM. Finally, we detected the absorbance changes of MB, Cu-MOF@CDDP (without GSH), and Cu-MOF@CDDP (with 10 mM GSH) using UV–vis spectroscopy. Each sample was subjected to three parallel tests.

### 3.7 Cu-MOF@CDDP mediated GSH deletion

We employed DTNB to evaluate the responsiveness of Cu-MOF@CDDP nanoparticles (NPs) to glutathione (GSH). Initially, we combined 240 μL of DTNB (2.5 mg/mL) in NC, Cu-MOF@CDDP (200 μg/mL), and 30 μL of a 10 mM GSH solution in deionized water. The final concentrations of Cu-MOF@CDDP were set at 0, 10, 20, 40, and 80 μg/mL. The mixture was then allowed to incubate at 25°Cfor 30 min, after which centrifugation was performed to separate the Cu-MOF@CDDP. Finally, the absorbance of the supernatant was measured by UV–vis spectroscopy. Each sample was tested in triplicate.

### 3.8 Cell culture

RKO and CT26 cells were used as the CRC cell model. We added 10 % FBS and 1 % antibiotics to the culture media of these two types of cells, and then placed them in a 37 °C incubator containing 5 % CO_2_ for cultivation.

### 3.9 The GSH detection in CRC cells

The GSH detection kit was used to study the Cu-MOF@CDDP mediated GSH depletion in CRC cells. First, we collected RKO cells and CT26 cells treated with Cu-MOF@CDDP NPs at concentrations of 0, 125, and 250 mg/mL for 4 h, and washed twice with NC. The cells were re-suspended in a reagent of three times volume as the sediment volume of the cells and then repeated freeze-thaw cycles 2–3 times. After centrifugation at 8000*g* for 15 min, the supernatant should be collected and stored at 4 °C. Finally, different samples will be treated with specific reagents following the manufacturer's protocol, followed by the addition of the substrate. Each group was performed to three parallel tests.

### 3.10 In vitro Anti-CRC effects of Cu-MOF@CDDP

RKO and CT26 cells were placed in 96-well plates at a density of 8000 cells/well and incubated for 24 h. Subsequently, the original culture media was replaced with fresh media containing CDDP, Cu-MOF, and Cu-MOF@CDDP, with the equivalent dosage of CDDP. After incubating the cells for 4 h, the culture media was discarded and the cells were washed 3 times with NC, followed by the addition of fresh culture media. These cells were continued to be cultured for an additional 2 d before determining cell viability using the standard MTT assay.

### 3.11 ***Intracellular OH***• ***detection***

We measured the levels of intracellular ROS according to the relevant literature. First, we placed RKO and CT26 cells in a 12-well plate at a density of 1.5 × 10^5^ cells per well and cultured them for 1 d. Once the cells had adhered to approximately 80 %, the original media was replaced with fresh medium containing NC, CDDP, Cu-MOF, and Cu-MOF@CDDP, with the equivalent dosage of Cu-MOF at 250 μg/mL, and continued to culture for an additional 6 h. Next, we washed the RKO and CT26 cells with NC and incubated them with DCFH-DA at 37 °C for 30 min. After that, the cells were collected in flow cytometry tubes following digestion with trypsin-EDTA solution. Finally, the above groups were recorded for fluorescence signals using a flow cytometer.

### 3.12 Animal models

Male BALB/c mice, aged 4–5 weeks, were obtained from GemPharmatech (Chengdu, China). The mice were xenografted with CT26 tumors by injecting 1 × 10^6^ CT26 cells subcutaneously into the right forelimb area of the BALB/c mice. The *in vivo* studies were carried out following the ethical guidelines for animal research set by Kunming Medical University.

### 3.13 In vivo Anti-CRC effect of Cu-MOF@CDDP

The mice with tumors were randomly assigned to four groups (n = 5 per group): NC, CDDP, Cu-MOF NPs, and Cu-MOF@CDDP NPs. A dose of 3 mg∙kg^−1^ of CDDP was administered intravenously to each group at two-day intervals. Subsequently, we monitored the body mass and tumor size of the mice, with the latter being determined using a specific formula:

$$\text{Tumor}\;\text{volume}=\frac{l\times s^{2}}{2}$$

The tumor dimensions were measured, with the longest and smallest diameters denoted as l and s, respectively. On the 18th day, the mice were killed, and tumors along with normal organs were harvested. Photographs and mass of tumor tissues from each group were taken. The tumor inhibition rate was then determined using the following formula:

$$Tumor\;inhibition\;ratio\left(\%\right)=\frac{W_{c}-W_{t}}{W_{c}}\times 100\%$$

In this formula, $W_{c}$ refers to the average tumor mass for the NC group, while $W_{t}$ indicates the ultimate tumor mass for the other groups.

### 3.14 Apoptosis analysis

RKO/CT26 cells were treated with CDDP, Cu-MOF, Cu-MOF@CDDP for 4 h. Subsequently, cell apoptosis detection kit was utilized to assess cell apoptosis, adhering to the manufacturer's guidelines.

### 3.15 The activation of cGAS-STING pathway

To examine the key proteins related with the STING signaling pathway in CRC cells (including STING, p-STING, IRF3, and p-IRF-3) after different treatments, western blot analysis was utilized. β-actin used as a control for protein loading. Moreover, to assess the stimulation of immune responses within the co-culture setup, flow cytometry was performed to evaluate the presence of DCs, Treg cells, CD4^+^ T cells, and CD8^+^ T cells. Furthermore, CT26 cells were incubated with the activated immune cells for 24 h.

### 3.16 Bilateral anti-tumor effects and immunotherapy of Cu-MOF@CDDP

8 × 10^5^ RKO/CT26 cells were injected subcutaneously into the right underarm (defined as the primary tumor) of male BALB/c mice. One week later, 4 × 10^5^ RKO/CT26 cells were similarly injected into the left underarm (defined as the metastatic tumor). The mice were then randomly assigned to five groups (n = 5): NC, CDDP, Cu-MOF, and Cu-MOF@CDDP, once the tumors reached approximately 100 mm^3^. The mice received an equal dose of CDDP (3 mg/kg) via intratumoral injection (only for in situ tumors) every two days for a total of 15 days. Tumor sizes and body weights were monitored daily. Following the specified treatments, the mice were euthanized, and the presence of DCs, Tregs, CD4^+^/CD8^+^ T cells in both the primary and metastatic tumors and spleens was assessed using flow cytometry.

### 3.17 Statistical analysis

All statistical analyses and graphics were used GraphPad Prism 8.0 software. All data were expressed as the mean ± standard deviation. Groups were compared using two-tailed Student's t-test or one-way ANOVA. The differences were considered significant for *p* values ∗ <0.05, ∗∗ <0.01, and ∗∗∗ <0.001.

## 4 Conclusions

In summary, we reported a repurposed copper-based metal-organic nano-frameworks (Cu-MOF@CDDP) for efficient suppression of cancer stemness and sensitization of chemo-immunotherapy. This repurposed Cu-MOF@CDDP was effectively internalized by CRC cells via endocytosis, releasing free copper ions and CDDP in a high-GSH environment. CDDP forms DNA-CDDP adducts, inhibiting DNA synthesis and repair, while Cu^2+^ induced cuproptosis by disrupting mitochondrial metabolism. DNA fragments from both the damaged nucleus and mitochondria activate the cGAS-STING pathway to provoke the anti-tumor immune response. Furthermore, Cu^2+^ depleted intracellular GSH and induced ROS accumulation for further triggering cuproptosis, resultimng in the downregulation of stemness-related proteins for enhancing chemo-immunotherapy efficacy. Therefore, Cu-MOF@CDDP demonstrated significant inhibitory effects on CRC proliferation and distant metastasis without notable toxicity or side effects on normal tissues, offering a novel strategy to potentiate chemo-immunotherapy.

## CRediT authorship contribution statement

**Yichun Huang:** Project administration. **Hailong Tian:** Project administration. **Zhimin Yue:** Investigation. **Lei liang:** Formal analysis. **Canhua Huang:** Funding acquisition. **Huili Zhu:** Writing – review & editing, Writing – original draft, Project administration. **Jun Yang:** Conceptualization.

## Ethics approval and consent to participate

All animal experiments conformed to the requirements of the institutional animal use and care system of Kunming Medical University.

## Declaration of competing interest

The authors declare that they have no known competing financial interests or personal relationships that could have appeared to influence the work reported in this paper.

## Data Availability

The authors do not have permission to share data.
