# Supplementary material for: Copper-coordination driven nano-frameworks for efficient colorectal cancer chemo-immunotherapy by suppression of cancer cell stemness
Source: Mater Today Bio. 2025 Mar 24;32:101707. doi: 10.1016/j.mtbio.2025.101707 (PMC11999347; doi:10.1016/j.mtbio.2025.101707)
Supplement: Multimedia component 1 [file mmc1.docx]

**Supporting Information**

**Copper-coordination Driven Nano-frameworks for Efficient Colorectal Cancer Chemo-immunotherapy by the Suppression of Cancer Cell Stemness**

Yichun Huang^a,1^, Hailong Tian^b,1^, Zhimin Yue^b,1^, Lei liang^a^, Canhua Huang^b,c^, Huili Zhu^d＊^, Jun Yang^a,＊^.

^a^ Department of Surgical Oncology, The First Affiliated Hospital of Kunming Medical University, Kunming, 650032, China

^b^ State Key Laboratory of Biotherapy and Cancer Center, West China Hospital, and Collaborative Innovation Center for Biotherapy, Chengdu, P.R. China.

^c^ Frontiers Medical Center, Tianfu Jincheng Laboratory, Chengdu, 610041, P.R. China.

^d^Department of Reproductive Medicine, Key Laboratory of Birth Defects and Related Diseases of Women and Children of Ministry of Education, West China Second University Hospital of Sichuan University, Chengdu, China.610041.

^1^These authors contributed equally to this work.

^＊^Corresponding authors.


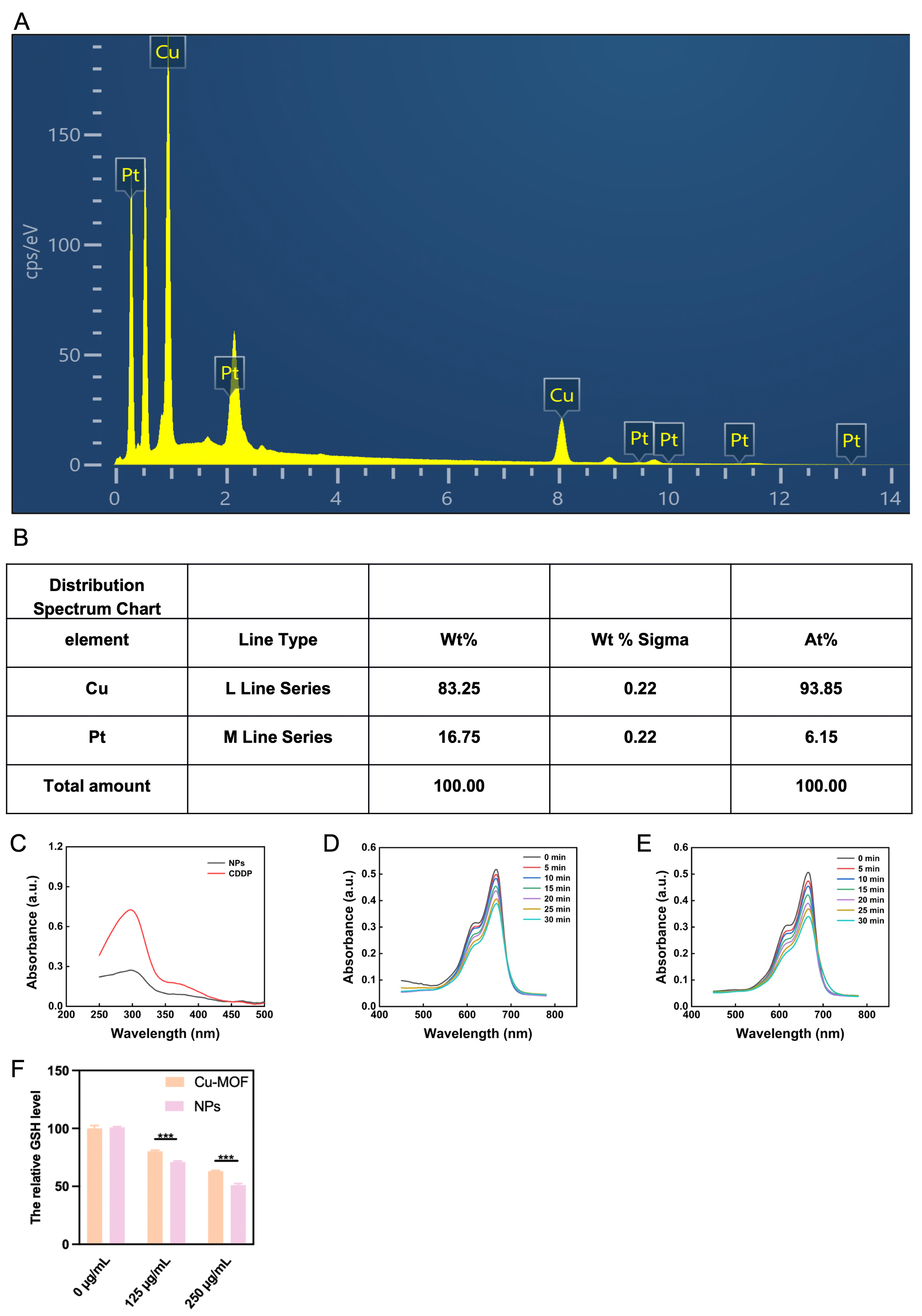


**Figure S1.** A) Element distribution of NPs. B) The distribution of copper and platinum elements Total spectrum. C) UV–vis absorption spectra of NPs and CDDP.

D) and E) Detection of hydroxyl radical generation by Cu-MOF and NPs (150µg/mL), respectively, utilizing MB as a molecular probe. F) Quantification of GSH depletion via reaction with DTNB, reflecting oxidative stress induction.


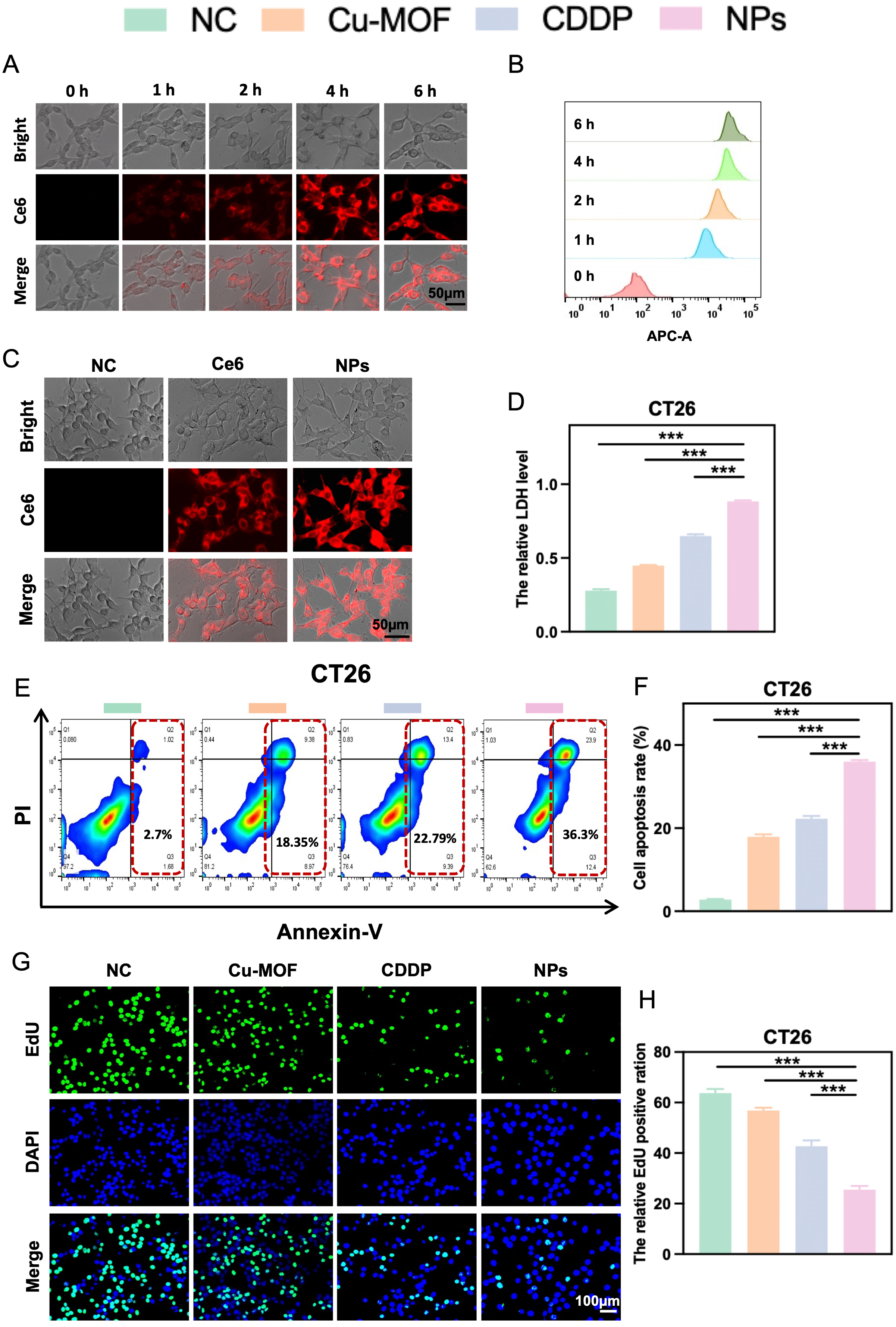


**Figure S2.** A) Fluorescence microscopy images and B) flow cytometry results of the cellular uptake of NPs in CT26 cells at different times. Scale bar: 50μm. C) Cellular uptake of NC, Ce6, NPs by CT26 cells following 4 h incubation. Scale bar: 50 μm. D) The relative LDH content analysis of CT26 cells. E) Flow cytometry plots and quantification results of apoptosis in CT26 cells. F) Quantitative results of cell apoptosis rate in CT26 cells. G) EdU assay of CT26 cells. Scale bar: 100 μm. H) Quantitative results of EdU assay in CT26 cells. The differences were considered significant for *p* values * < 0.05, ** < 0.01, and *** < 0.001.


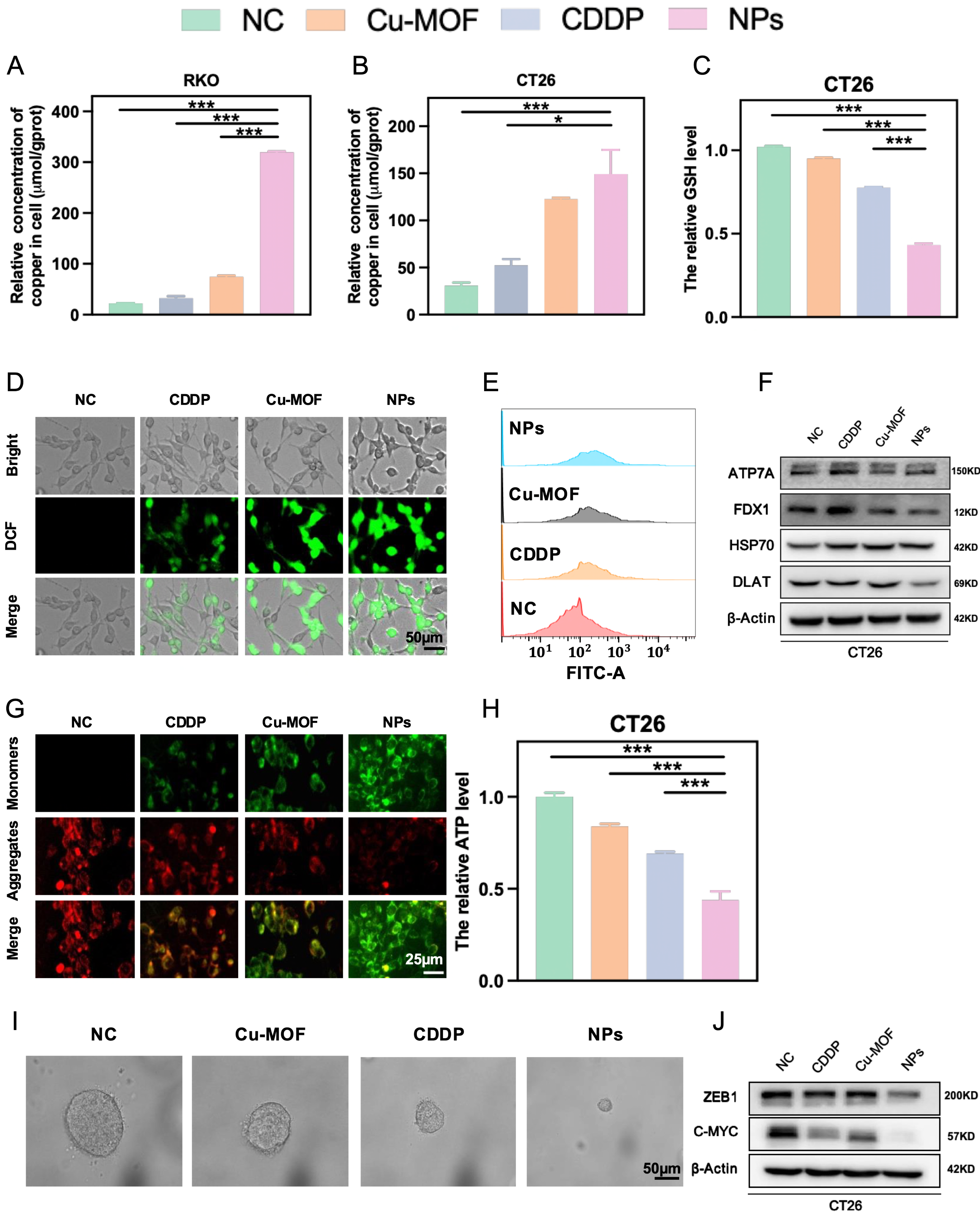


**Figure S3**. The relative concentration of cooper in cells of (A) RKO and (B) CT26 cells. C) Relative GSH content analysis of CT26 cells. D) Fluorescence imaging of intracellular ROS levels in CT26 cells using DCFH-DA as a probe. Scale bar: 50 μm. E) low analysis for intracellular ROS generation using DCFH-DA probe. F) Western blot analysis of ATP7A, FDX1, HSP70, DLAT expression in CT26 cells following various treatments. G) Mitochondrial membrane potentials of CT26 cells were determined by JC-1 assay after different treatments. Scale bar: 25 μm. H) Relative ATP content analysis of CT26 cells. I) Sphere-formation assay results for CT26 cells after different treatments. Scale bar: 50 μm. J) Western blot analysis of ZEB1, c-MYC expression in CT26 cells following various treatments. The differences were considered significant for *p* values * < 0.05, ** < 0.01, and *** < 0.001.


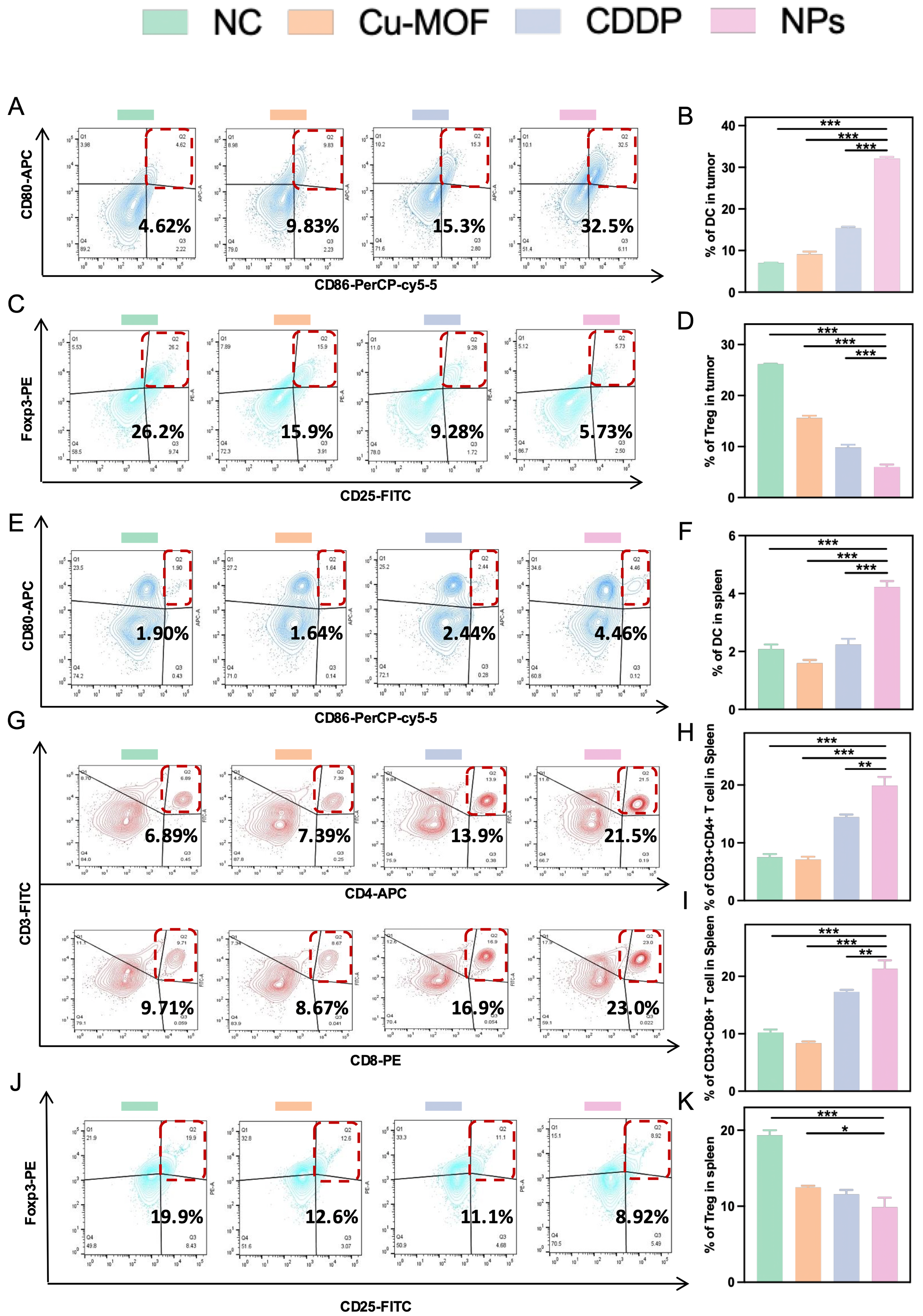


**Figure S4.** Flow cytometry plots and quantification of A-B) DCs, C-D) Tregs in tumor tissues. Flow cytometry plots and quantification of E-F) DCs, G-I) CD3^+^CD4^+^/CD3^+^CD8^+^ T cells and J-K) Tregs in spleens. The differences were considered significant for *p* values * < 0.05, ** < 0.01, and *** < 0.001.


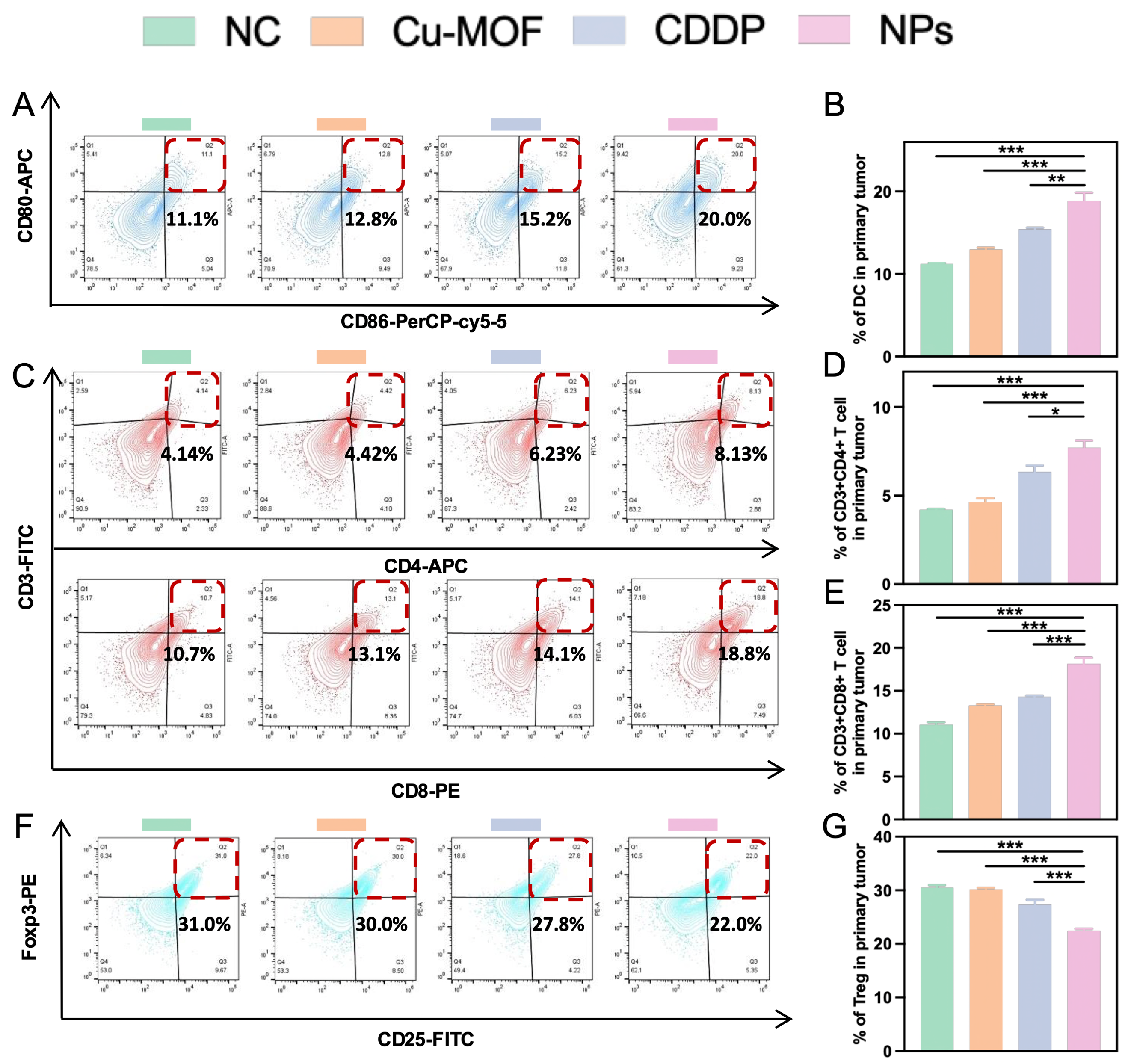


**Figure S5.** Flow cytometry plots and quantification results of A-B) DCs, C-E) CD3^+^CD4^+^/CD3^+^CD8^+^ T cells, and F-G) Tregs. The differences were considered significant for *p* values * < 0.05, ** < 0.01, and *** < 0.001.


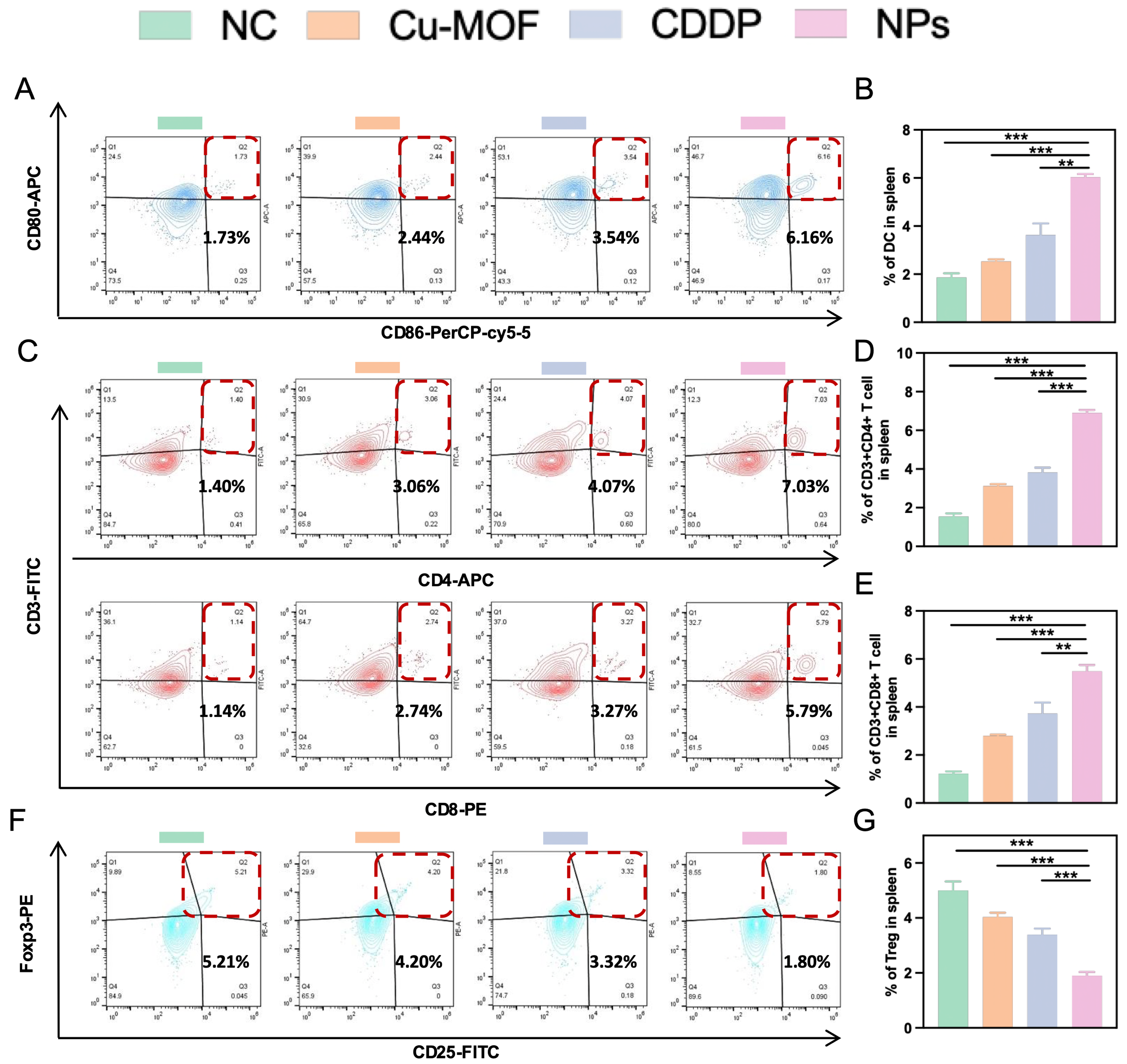


**Figure S6.** Flow cytometry plots and quantification of A-B) dendritic cells (DCs), C-E) cytotoxic T cells (CD3^+^CD4^+^/CD3^+^CD8^+^), and F-G) regulatory T cells (Tregs). The differences were considered significant for *p* values * < 0.05, ** < 0.01, and *** < 0.001.


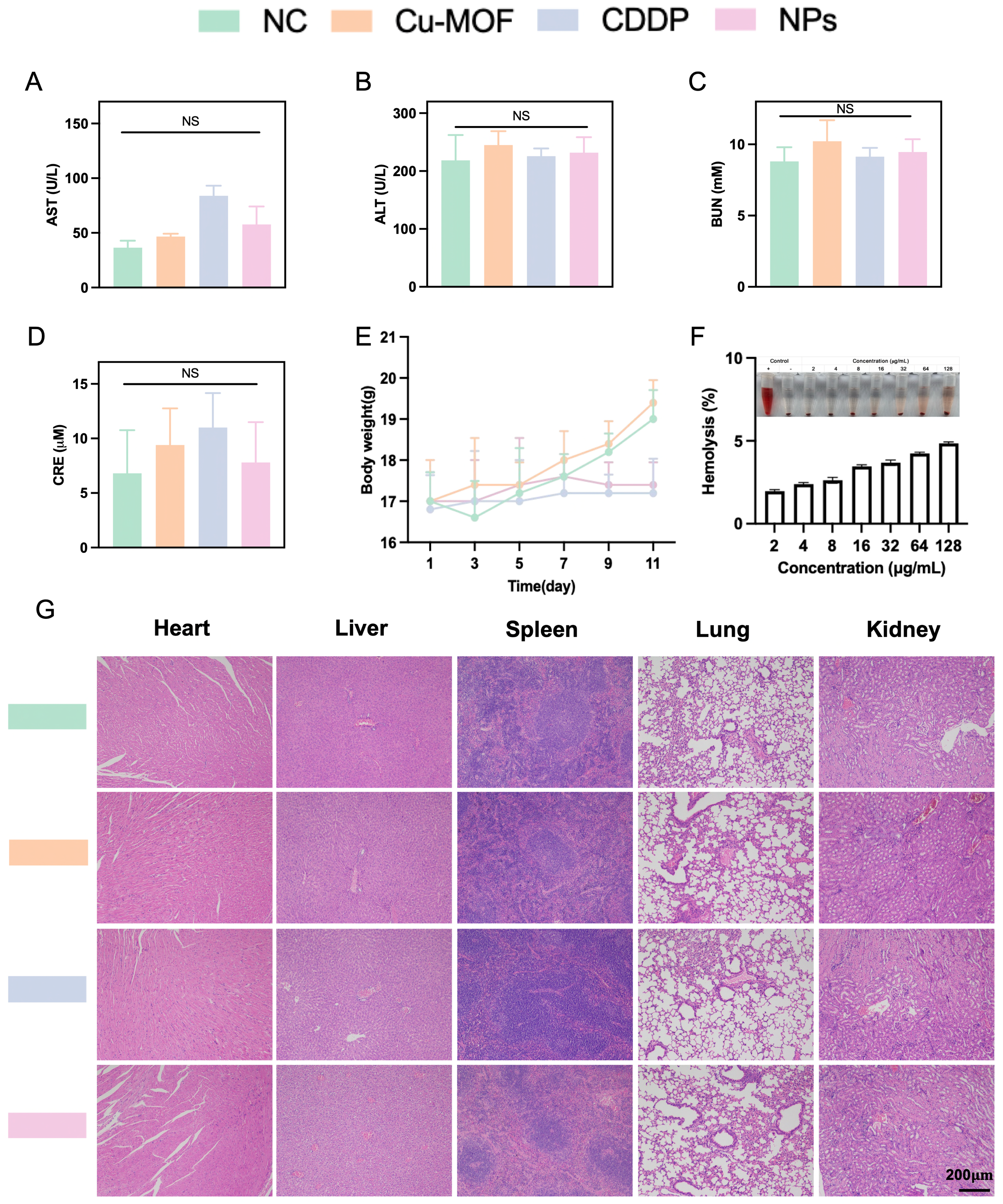


**Figure S7.** A) Relative AST content analysis of RKO cells. B) Relative ALT content analysis of RKO cells. C) Relative BUN content analysis of RKO cells. D) Relative CRE content analysis of RKO cells. E) Body weight change curves (n = 5). F) The hemolysis rate of the nanoformulation. G) Histopathological assessment of vital organs, including cardiac, hepatic, splenic, pulmonary, and renal tissues. Scale bar: 200 μm. The differences were considered significant for *p* values * < 0.05, ** < 0.01, and *** < 0.001.
